# Chewing Prevents Stress-Induced Hippocampal LTD Formation and Anxiety-Related Behaviors: A Possible Role of the Dopaminergic System

**DOI:** 10.1155/2015/294068

**Published:** 2015-05-17

**Authors:** Yumie Ono, So Koizumi, Minoru Onozuka

**Affiliations:** ^1^Health Science and Medical Engineering Laboratory, School of Science and Technology, Meiji University, Room A806, 1-1-1 Higashi-Mita, Tama-ku, Kawasaki, Kanagawa 214-8571, Japan; ^2^Orthodontic Division, Department of Oral Science, Kanagawa Dental University Graduate School, 82 Inaoka-cho, Yokosuka, Kanagawa 238-8580, Japan; ^3^Department of Judo Therapy and Medical Science, Faculty of Medical Science, Nippon Sport Science University, 1221-1 Kamoshida-cho, Aoba-ku, Yokohama 227-0033, Japan

## Abstract

The present study examined the effects of chewing on stress-induced long-term depression (LTD) and anxiogenic behavior. Experiments were performed in adult male rats under three conditions: restraint stress condition, voluntary chewing condition during stress, and control condition without any treatments except handling. Chewing ameliorated LTD development in the hippocampal CA1 region. It also counteracted the stress-suppressed number of entries to the center region of the open field when they were tested immediately, 30 min, or 60 min after restraint. At the latter two poststress time periods, chewing during restraint significantly increased the number of times of open arm entries in the elevated plus maze, when compared with those without chewing. The *in vivo* microdialysis further revealed that extracellular dopamine concentration in the ventral hippocampus, which is involved in anxiety-related behavior, was significantly greater in chewing rats than in those without chewing from 30 to 105 min after stress exposure. Development of LTD and anxiolytic effects ameliorated by chewing were counteracted by administering the D1 dopamine receptor antagonist SCH23390, which suggested that chewing may activate the dopaminergic system in the ventral hippocampus to suppress stress-induced anxiogenic behavior.

## 1. Introduction

Chewing has been known as one of the active coping strategies to suppress stress. We have previously shown that chewing during stress exposure significantly attenuates neuronal responses to stress and the subsequent stress-related cognitive deficits in the hippocampus, such as impairment of spatial memory [1, 2]. Stress-induced changes in neuronal electrical properties in the hippocampus not only contribute to memory functions, but also mediate anxiety behavior [3, 4]; the hippocampus is a key region to express stress- and anxiety-related behaviors* via* its reciprocal connection with the amygdala and the prefrontal cortex [5]. In addition to its possible role in memory formation with counterpart phenomenon, long-term potentiation, hippocampal long-term depression (LTD) has been suggested to have a correlation with anxiety behavior [6]. Therefore, it is intriguing to investigate whether chewing under stressful conditions could interfere with stress-induced LTD and corresponding anxiety-like behavior. The present electrophysiological study evaluated the effects of spontaneous chewing during exposure to restraint stress on stress-related LTD development in the rat hippocampal CA1 region. We also used two measures of anxiety-like behaviors, exploration in a novel open field and an elevated plus maze, to determine time-course changes in stress-induced anxiety-like behavior in rats that were stressed with or without coping. We also incorporated* in vivo* microdialysis analysis to measure monoaminergic signals in the hippocampus that relate to different anxiety-like behaviors found in rats depending on their coping styles [7]. Finally, we tested whether the pharmacological inhibition of the dopaminergic D1 receptor could counteract the effect of chewing in hippocampal LTD and in anxiety-like behavior. These results helped to determine the possible role of the dopaminergic signaling pathway in the mechanism of how chewing interacts with hippocampal synaptic plasticity to suppress stress-induced anxiety-like behavior.

## 2. Materials and Methods

### 2.1. Animals

Ten-week-old male Sprague-Dawley rats were maintained in a temperature-controlled room (22 ± 3°C) with a 12 h light/dark cycle (lights on at 7:00 a.m.). The rats had free access to water and food. All experiments were in accordance with the guidelines for Animal Experimentation of Kanagawa Dental University. All efforts were made to minimize the number of animals used and their suffering.

### 2.2. Stress Protocol and Drug Administration

To produce restraint stress, we tied a rat to a wooden board in a spread-eagle supine position for 30 min using leg fasteners as previously described [8]. Rats were randomly assigned to one of the three conditions of (1) stressed (ST), (2) stressed and chewing (SC), and (3) control (CT). Rats in group ST were restrained and left alone for the entire restraint period, and those in group SC were allowed to chew on a wooden stick (diameter, 5 mm) that was placed near the animal's mouth during restraint. Every rat in group SC responded to the wooden stick by chewing on it with a rapid and repetitive sequence of jaw opening and closing movements for at least two-thirds of the total restraint period. Rats in group CT were handled in the same manner as those in group ST and group SC but were returned to their home cage for 30 min instead of restraint. Some of the rats in group SC were administered intraperitoneal injections of 0.3 mg/kg SCH23390, a selective dopaminergic D1-receptor antagonist that penetrates the blood-brain barrier [9], which was dissolved in 0.9% saline 15 min prior to the stress protocol. All stress inducement and handling manipulations were performed between 9:00 a.m. and 11:00 a.m.

### 2.3. Electrophysiology

A total of 21 rats were used in the LTD experiment. Six rats were assigned to each group of CT, ST, and SC, respectively, and the remaining three rats were assigned to group SC+SCH23390. Immediately after the stress protocol, we anesthetized rats with 2-bromo-2-chloro-1,1,1-trifluoroethane (300 *μ*L/100 g; Takeda Chemical Industries, Osaka, Japan), decapitated them, quickly removed their brains, and iced them in artificial cerebrospinal fluid (ACSF) containing 119 NaCl, 2.5 KCl, 26.2 NaHCO_3_, 1 NaH_2_PO_4_, 1.3 MgSO_4_, 2.5 CaCl_2_, and 11 glucose (in mM, bubbled with 95% O_2_–5% CO_2_). We dissected the hippocampi, embedded them in agar blocks for slicing, cut transverse slices (450 *μ*m thick) with a vibrating tissue slicer (Dosaka, Kyoto, Japan), and transferred them to a holding chamber at room temperature (25°C). We allowed the slices to recover for at least 60 min and then transferred them to an immersion-type recording chamber perfused at 1 mL/min with ACSF containing 0.1 mM picrotoxin (Sigma-Aldrich, Tokyo, Japan) at room temperature. To prevent epileptiform discharge of pyramidal neurons, we made a cut at the border between the CA1 and CA3 areas. A glass pipette filled with 3 M NaCl and positioned in the stratum radiatum of the CA1 area recorded the field excitatory postsynaptic potential (fEPSP). Bipolar stainless-steel electrodes (World Precision Instruments, Sarasota, FL, USA) placed in the stratum radiatum on opposite sides of the recording pipette stimulated the Schaffer collateral branches. We adjusted the intensity of fEPSP in the baseline period to around 50% of the maximal response and then recorded stable baseline fEPSP activity by applying a 40 *μ*s voltage pulse at the determined intensity every 30 s for at least 10 min. LTD was induced by low-frequency stimulation (LFS) of 900 pluses at 1 Hz (15 min). All signals were filtered at 2 kHz using a low-pass Bessel filter and digitized at 5 kHz using a MultiClamp 700A interface running pCLAMP software (Axon Instruments, Union City, CA, USA). We measured initial slopes of the fEPSP and normalized them to the average of the values measured over the baseline period. The average slope size of the fEPSPs recorded between 30 and 40 min after the end of the LFS provided the basis for our statistical comparisons. We used a single slice from a single animal for subsequent analysis.

### 2.4. Open-Field Test (OFT) and Elevated Plus Maze Test (EPT)

Eighty-nine and 87 rats were used in the OFT and EPT experiments, respectively. OFT and EPT were performed in a sound-isolated room to analyze anxiety-related behavior in a novel environment [10]. The open field consisted of a circular arena (150 cm in diameter) surrounded by walls 40 cm in height. A video camera (DCR-HC1000, Sony, Tokyo, Japan) was suspended from the ceiling above the arena to observe and record animal behavior. The field was constantly illuminated with an intense light (600 Lx at the floor of the arena). The animals in their home cage were brought into the experimental room at least 1 h before the beginning of the first trial of the day to acclimatize.

At the beginning of the OFT, the rat was placed at the center of the arena. The field was divided into two subdivisions of center (75 cm in diameter) and peripheral areas for scoring ambulatory activity. Rats were allowed to freely explore the arena for 5 min. The number of entrances into the subdivisions was recorded using a video tracking system (Top Scan, Clever Sys, Reston, VA, USA) as parameters for OFT. An entrance to a subdivision was counted if the center of gravity of the rat body passed through the border of the subdivision from outside to inside. After the completion of each trial, the animal was returned to the home cage and the field was cleaned with 30% ethanol.

The elevated plus maze had dimension of 1,100 mm in width and length. The corridors were 500 mm in length and 100 mm in width, and everything was raised 500 mm above the floor. Two facing corridors were closed by walls 450 mm in height (closed arms), and the rest remained open (open arms). The rat was initially placed at the center of the maze facing the open arm and was allowed to freely explore the maze for 5 min. The number of entrances to the open arm was measured for scoring anxiety behavior. The entrance to the open arm was counted if the center of gravity of the rat body passed through the border of the open arm from the center of the maze. After completion of each trial, the animal was returned to the home cage and the field was cleaned with 30% ethanol.

Rats were tested in either OFT or EPM at 0, 30, and 60 min after stress exposure. Fifty-one rats were administered SCH23390 and tested at 60 min after stress exposure.

### 2.5. Microdialysis

Concentrations of norepinephrine (NE), dopamine (DA), and serotonin (5-HT) were determined in the right ventral hippocampus (5.0 mm posterior, 5.0 mm lateral, and 3.0 mm inferior from the bregma) and were measured in an additional group of 15 rats. The ventral hippocampus was chosen because of its specific role in regulating anxiety-related behavior [11]. These rats were first implanted with a dialysis guide cannula with a dummy probe and divided into two subgroups that were treated in an identical fashion to the ST (*n* = 7) and SC (*n* = 8) groups after recovery of 4–6 days. Food and water were freely provided by the time of microdialysis measurement, and the rats were housed individually after probe implantation. On the day of stress exposure, a dialysis probe (A-I-8-02, Eicom, Kyoto, Japan) was inserted into the guide cannula instead of the dummy probe. The dialysis tube was directly connected to a high-performance liquid chromatography apparatus (Eicom, Kyoto, Japan) for online analysis of NE, DA, and 5-HT. A microperfusion pump perfused the hippocampus with normal Ringer's solution (147 mM NaCl, 4 mM KCl, 2.3 mM CaCl_2_) through the dialysis tube at a flow rate of 1 mL/min. The dialysis sample was injected every 15 min* via* auto-injector (EAS-20, Eicom, Kyoto, Japan). The mobile phase consisted of 99% 0.1 M sodium phosphate-buffered solution, 1% methanol, 500 mg/L sodium 1-decanesulfonate, and 50 mg/L Na_2_-EDTA. NE, DA, and 5-HT were separated on a 30 mm × 4.6 mm diameter Eicompack CAX column at 35°C. The working electrode (HTEC-500; Eicom, Kyoto, Japan) was composed of graphite, and the flow rate was set at 250 mL/min. Concentrations of NE, DA, and 5-HT were measured using known concentrations of the corresponding standard, which were quantified by means of the peak area ratio. All chemicals and drugs used as corresponding standards and the internal standard were purchased from Sigma (Tokyo, Japan). Chromatograms were obtained using the appropriate software (Power Chrom version 2; eDAQ Pty.).

### 2.6. Statistics

All values shown are mean ± S.E.M. Statistical analysis was conducted using IBM SPSS Statistics 21 (IBM, Armonk, NY, USA) and R [12]. We compared values using the one-way analysis of variance (ANOVA) test with post hoc Tukey's multiple comparisons or the Kruskal-Wallis test and the following pairwise comparisons depending on normality of the data. Because microdialysis data were repeated measures from animals in either condition of ST of SC, a two-way ANOVA with repeated measures and the post hoc multiple comparisons with Bonferroni correction were applied. We consider *P* values < 0.05 to be statistically significant, unless otherwise stated.

## 3. Results

### 3.1. Chewing Rescued Stress-Related LTD

**Figure 1:**
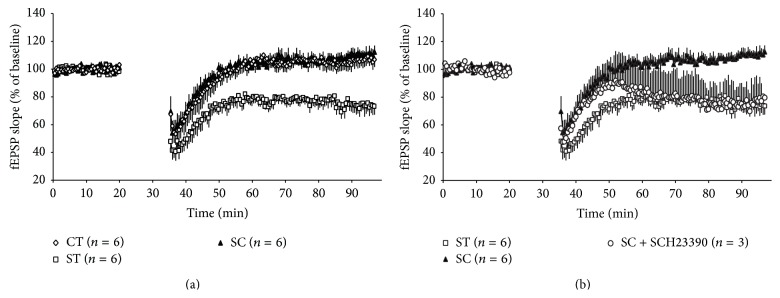
Effects of acute stress and SCH23390 on hippocampal CA1 LTD. (a) Time course of mean fEPSP slopes for different experimental groups. CT, ST, and SC refer to rats in the control, stressed, and stressed-with-chewing groups, respectively, without SCH23390 administration. LFS was applied during 20–35 min to induce LTD. (b) Time course of mean fEPSP slopes for SCH23390-treated SC groups (SC+SCH23390). Data from groups ST and SC were taken from (a) and superimposed for the comparison.

In agreement with earlier findings [6, 13], LTD was not induced in control rats, but in stressed rats (Figure 1(a)). Chewing rescued stress-induced formation of LTD. The average slope size of fEPSPs was significantly suppressed in group ST (77.9 ± 3.3%) compared with group CT (105.2 ± 4.6%) and group SC (106.4 ± 4.8%; *F*(2,17) = 14.11, *P* < 0.001). These results suggested an ameliorative effect of chewing on stress-related induction of LTD in the adult hippocampus. Administration of SCH23390 counteracted the effect of chewing in group SC. Blockage of dopaminergic neurotransmission during stress exposure and chewing decreased a late phase of fEPSP slope and induced LTD (Figure 1(b)).

### 3.2. Chewing Rescued Anxiety Behavior and Facilitated Poststress Exploratory Activity

**Figure 2:**
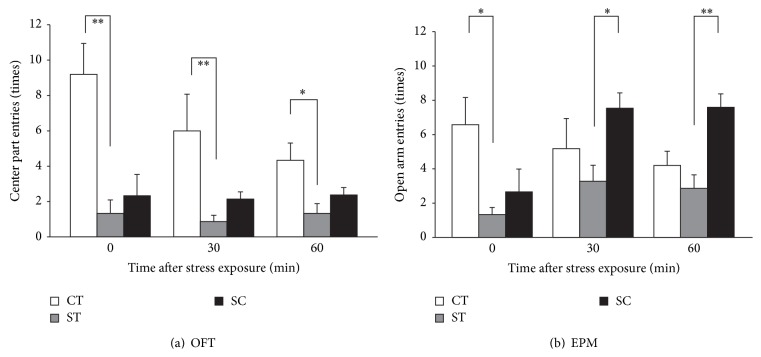
Effects of acute stress and chewing on poststress exploratory behavior in different durations after stress exposure. (a) The number of entries to the center region of the OFT. The number of animals used was *n* = (0 min: 5, 6, 6), (30 min: 5, 8, 7), and (60 min: 9, 9, 8) after stress exposure in the (CT, ST, and SC) groups, respectively. (b) Number of entries to the open arm of the EPM. The number of animals used was *n* = (0 min: 5, 6, 6), (30 min: 5, 8, 7), and (60 min: 9, 8, 8) after stress exposure in the (CT, ST, and SC) groups, respectively. Asterisks indicate statistically significant differences (^∗^ *P* < 0.05, ^∗∗^ *P* < 0.01).

OFT results (Figure 2(a)) showed that restraint stress significantly reduced the number of entries to the center region in group ST (1.3 ± 0.8 times; *P* < 0.017), but not in group SC (2.3 ± 1.2 times), compared with group CT (9.2 ± 1.7 times; Kruskal-Wallis chi-squared = 8.90) immediately after stress. The suppressed number of entries to the center region in group ST lasted for at least 1 h (0.9 ± 0.4 and 1.3 ± 0.6 times at 30 and 60 min from stress exposure, resp., *P* = 0.005 and 0.026 in group ST, resp.). These results indicated acute restraint stress suppressed exploratory activity, which was counteracted by active coping with chewing.

The number of open arm entries in the EPM (Figure 2(b)) also demonstrated a stress-related decrease in the number of entries to the open arm in group ST (1.3 ± 0.4 times; *P* = 0.02) but not in group SC (2.7 ± 1.3 times), compared with group CT (6.6 ± 1.6 times; *F*(2,16) = 5.10, *P* = 0.022) immediately after stress. However, the suppressed number of entries to the open arm recovered at 30 min after stress exposure. Interestingly, group SC rats exhibited more open arm entries after stress exposure compared with group ST at 30 min (7.8 ± 0.9 versus 3.3 ± 0.9 times; *P* < 0.034) and at 60 min (7.6 ± 0.8 versus 2.9 ± 0.8 times; *P* = 0.006) after stress. Note that the mean number of open arm entries in group SC was larger than in group CT in these two poststress timings, although they did not reach statistical significance. These results suggest an anxiolytic effect of chewing under stress.

### 3.3. Chewing Increased Poststress Hippocampal DA Concentrations

**Figure 3:**
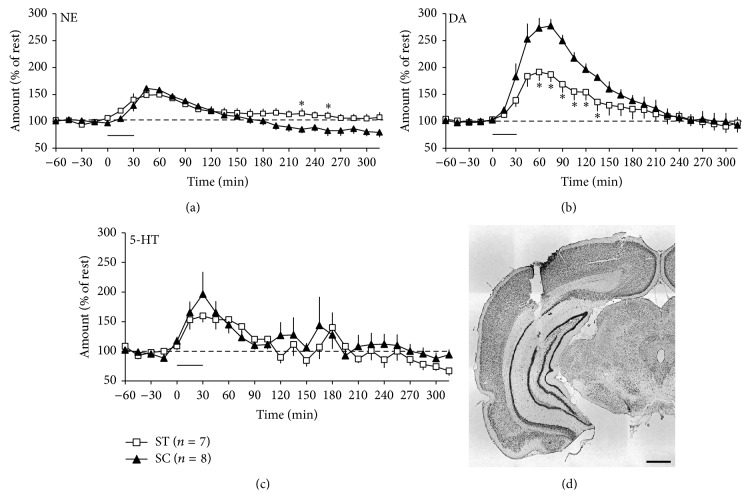
Effects of acute stress and chewing on concentration changes of norepinephrine (a), dopamine (b), and serotonin (c) in the ventral hippocampus. The thick horizontal bar indicates exposure to restraint stress. Asterisks indicate statistically significant differences (*P* < 0.05). (d) A representative brain slice showing the tract of microdialysis probe. Postmortem brain slices of all tested rats were Nissl stained to confirm the microdialysis tract in the ventral hippocampus and were analyzed using a light microscope. Scale bar equals 1 mm.

There was a statistically significant interaction between time and group in the time-course change of NE and DA concentrations in the ventral hippocampus, but not in 5-HT concentrations. The post hoc multiple comparisons found significant differences between groups ST and SC in NE concentrations at 195 and 225 min after stress exposure, respectively (Figure 3(a)), and those in DA concentrations from 30 to 105 min after stress exposure, respectively (Figure 3(b)). The time in which DA concentration significantly increased in group SC overlapped with the time in which rats from the same group exhibited significantly increased exploratory behavior in the EPM (30 and 60 min after stress exposure, resp.).

### 3.4. Antagonizing DA Transmission Inhibited the Anxiolytic Effect of Chewing

**Figure 4:**
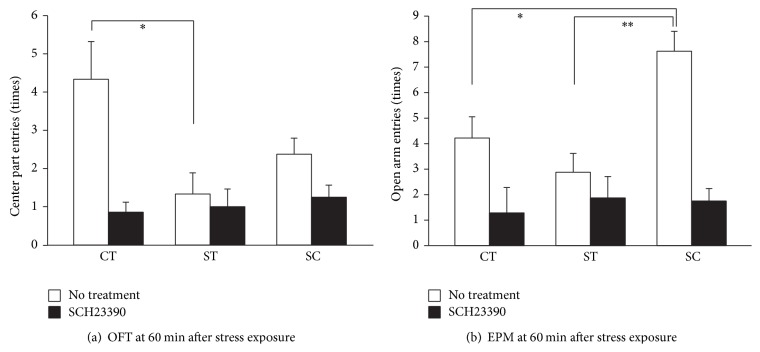
Effect of SCH23390 on exploratory behavior at 60 min after stress exposure. The number of animals administered SCH23390 (0.3 mg/kg i.p.) was *n* = (9, 9, 8) in the OFT and (9, 8, 8) in the EPM for (CT, ST, and SC) groups, respectively. Data of “no treatment” were taken from Figure 2 and shown for comparison. Asterisks indicate statistically significant differences (^∗^ *P* < 0.05, ^∗∗^ *P* < 0.01).

The synchronous increase in hippocampal DA concentrations and exploratory behavior further motivated us to investigate the role of dopaminergic transmission on the anxiolytic effect of chewing. As expected, systemic administration of SCH23390 suppressed the effect of chewing during exploratory activity at 60 min after stress exposure (Figure 4). There was no statistically significant difference among groups in the number of entries to the center region of the OFT and in those to the open arm in the EPM.

## 4. Discussion

The key finding of the present study is that active coping to restraint stress by chewing prevented stress-induced LTD in the adult male hippocampus, as well as poststress anxiety-like behavior. The development of the anxiolytic effect by chewing coincided with increased DA concentrations in the ventral hippocampus, which plays an important role in regulating anxiety-related behavior [11]. Pharmacological blockage of the dopaminergic D1-receptor counteracted the effect of chewing, both on LTD and on anxiety behaviors. These results suggest an ameliorative effect of chewing on stress-induced anxiety-like behavior* via* activation of the hippocampal dopaminergic signaling pathway.

Yang et al. [14] reported that stress facilitates LTD induction in adult rats, which is mediated through the activation of glucocorticoid receptors to affect extrasynaptic NR2B-containing NMDARs to undergo the induction of LTD. The present results confirmed the effect of stress to induce hippocampal LTD in adult rats (Figure 1(a)). Mockett et al. [15] further demonstrated that dopamine D1/D5 receptor activation counteracts NMDAR-dependent LTD, which is also consistent with our results showing that blockade of D1-receptor activation impaired the effect of chewing in rats that normally chewed during stress exposure (Figure 1(b)). The present results suggest that both a reduction in glucocorticoid plasma concentration [16] and facilitation of neuronal DA concentrations in the hippocampus are essential to prevent hippocampal LTD development in rats that chewed during stress exposure.

Our previous studies in rats demonstrated that chewing relieves stress by suppressing stress responses in endocrine and autonomic nervous systems. Active chewing during stress exposure suppresses metabolic activity in the hypothalamus [16], the higher control center of systemic stress response, to prevent stress-related secretion of corticosterone and norepinephrine in plasma [8, 16, 17]. We have also previously found a possible involvement of histaminergic neuronal pathways to rescue stress-suppressed long-term potentiation in hippocampal neurons [18]. The current study further added a novel role of chewing in preventing stress-related anxiety behavior, possibly mediated by facilitating dopaminergic systems. The accumulating results suggest that chewing involves both direct hormonal effects and indirect neuronal mechanisms of the neural monoaminergic system to rescue NMDAR functions.

The dopaminergic system is essentially involved in rhythmic movement, including mastication [19, 20]. Reduced masticatory activity by molar extraction or powder-diet feeding reduces the response of hippocampal DA neurons, impairing hippocampal learning ability in the step-through passive avoidance test [21] and in the novel-object recognition test [22]. Results from the present study using microdialysis further demonstrated that active mastication facilitates DA release in the hippocampus (Figure 3). These results suggest a possible interaction between masticatory function and cognitive function in the hippocampus* via* modulation of DA responses. The late development of LTD in SCH23390-treated rats in group SC (Figure 1(b)) also supports the involvement of the DA pathway, because dopaminergic projection is essential for settlement of long-term plasticity in the hippocampus [23].

A limitation of this study was that we did not specifically block D1 receptors in the ventral hippocampus but used systemic administration of D1 receptor antagonists, which may act in different regions of the brain and affect exploratory behavior. These results were consistent with a previous report that systemic D1-antagonism reduces spatial exploration in a novel environment [9]. However, it would not affect the interpretation of the results that increased DA concentration in the ventral hippocampus by chewing is critical for preventing anxiety-like behavior, because intra-ventral hippocampal administration of SCH23390 itself has no modulatory effect on exploratory behavior and anxiety-like behavior [24, 25]. Further research is required to resolve the above methodological interference and confirm the results that we found in the current study.

## 5. Conclusions

Chewing during stress exposure could be an active coping strategy to relieve stress-induced anxiety-like behavior in rats. Behavioral examinations in various poststress time periods demonstrated a significant anxiolytic effect with chewing in stressed rats at 30 and 60 min after stress exposure, which corresponds with enhanced dopamine release in the ventral hippocampus. These results indicate possible involvement of the dopaminergic neuronal pathway in the stress-relieving mechanism of chewing.

## References

[1] Ono Y., Yamamoto T., Kubo K.-Y., Onozuka M. (2010). Occlusion and brain function: mastication as a prevention of cognitive dysfunction. *Journal of Oral Rehabilitation*.

[2] Miyake S., Yoshikawa G., Yamada K. (2012). Chewing ameliorates stress-induced suppression of spatial memory by increasing glucocorticoid receptor expression in the hippocampus. *Brain Research*.

[3] Kim J. J., Foy M. R., Thompson R. F. (1996). Behavioral stress modifies hippocampal plasticity through N-methyl-D-aspartate receptor activation. *Proceedings of the National Academy of Sciences of the United States of America*.

[4] Ma W.-P., Cao J., Tian M. (2007). Exposure to chronic constant light impairs spatial memory and influences long-term depression in rats. *Neuroscience Research*.

[5] Leuner B., Shors T. J. (2013). Stress, anxiety, and dendritic spines: what are the connections?. *Neuroscience*.

[6] Martinowich K., Schloesser R. J., Lu Y. (2012). Roles of p75 NTR, long-term depression, and cholinergic transmission in anxiety and acute stress coping. *Biological Psychiatry*.

[7] Zhukov D. A., Vinogradova K. P. (2002). Learned helplessness or learned inactivity after inescapable stress? Interpretation depends on coping styles. *Integrative Physiological and Behavioral Science*.

[8] Ono Y., Kataoka T., Miyake S. (2008). Chewing ameliorates stress-induced suppression of hippocampal long-term potentiation. *Neuroscience*.

[9] Clausen B., Schachtman T. R., Mark L. T., Reinholdt M., Christoffersen G. R. J. (2011). Impairments of exploration and memory after systemic or prelimbic D1-receptor antagonism in rats. *Behavioural Brain Research*.

[10] Carola V., D'Olimpio F., Brunamonti E., Mangia F., Renzi P. (2002). Evaluation of the elevated plus-maze and open-field tests for the assessment of anxiety-related behaviour in inbred mice. *Behavioural Brain Research*.

[11] Kjelstrup K. G., Tuvnes F. A., Steffenach H.-A., Murison R., Moser E. I., Moser M.-B. (2002). Reduced fear expression after lesions of the ventral hippocampus. *Proceedings of the National Academy of Sciences of the United States of America*.

[12] R Development Core Team (2011). *R: A Language and Environment for Statistical Computing*.

[13] Wagner J. J., Alger B. E. (1995). GABAergic and developmental influences on homosynaptic LTD and depotentiation in rat hippocampus. *Journal of Neuroscience*.

[14] Yang C.-H., Huang C.-C., Hsu K.-S. (2005). Behavioral stress enhances hippocampal CA1 long-term depression through the blockade of the glutamate uptake. *The Journal of Neuroscience*.

[15] Mockett B. G., Guévremont D., Williams J. M., Abraham W. C. (2007). Dopamine D1/D5 receptor activation reverses NMDA receptor-dependent long-term depression in rat hippocampus. *Journal of Neuroscience*.

[16] Ono Y., Lin H.-C., Tzen K.-Y. (2012). Active coping with stress suppresses glucose metabolism in the rat hypothalamus. *Stress*.

[17] Koizumi S., Minamisawa S., Sasaguri K., Onozuka M., Sato S., Ono Y. (2011). Chewing reduces sympathetic nervous response to stress and prevents poststress arrhythmias in rats. *American Journal of Physiology—Heart and Circulatory Physiology*.

[18] Ono Y., Kataoka T., Miyake S., Sasaguri K., Sato S., Onozuka M. (2009). Chewing rescues stress-suppressed hippocampal long-term potentiation via activation of histamine H1 receptor. *Neuroscience Research*.

[19] Chandler S. H., Goldberg L. J. (1984). Differentiation of the neural pathways mediating cortically induced and dopaminergic activation of the central pattern generator (CPG) for rhythmical jaw movements in the anesthetized guinea pig. *Brain Research*.

[20] Lapointe N. P., Rouleau P., Ung R.-V., Guertin P. A. (2009). Specific role of dopamine D1 receptors in spinal network activation and rhythmic movement induction in vertebrates. *The Journal of Physiology*.

[21] Kushida S., Kimoto K., Hori N. (2008). Soft-diet feeding decreases dopamine release and impairs aversion learning in Alzheimer model rats. *Neuroscience Letters*.

[22] Kawahata M., Ono Y., Ohno A., Kawamoto S., Kimoto K., Onozuka M. (2014). Loss of molars early in life develops behavioral lateralization and impairs hippocampus-dependent recognition memory. *BMC Neuroscience*.

[23] Lisman J. E., Grace A. A. (2005). The hippocampal-VTA loop: controlling the entry of information into long-term memory. *Neuron*.

[24] Nasehi M., Kafi F., Zarrindast M.-R. (2013). Differential mechanisms of opioidergic and dopaminergic systems of the ventral hippocampus (CA_3_) in anxiolytic-like behaviors induced by cholestasis in mice. *European Journal of Pharmacology*.

[25] Zarrindast M. R., Naghdi-Sedeh N., Nasehi M., Sahraei H., Bahrami F., Asadi F. (2010). The effects of dopaminergic drugs in the ventral hippocampus of rats in the nicotine-induced anxiogenic-like response. *Neuroscience Letters*.

